# Polyethylenimine-based nanocarriers in co-delivery of drug and gene: a developing horizon

**DOI:** 10.1080/20022727.2018.1488497

**Published:** 2018-07-03

**Authors:** Abbas Zakeri, Mohammad Amin Jadidi Kouhbanani, Nasrin Beheshtkhoo, Vahid Beigi, Seyyed Mojtaba Mousavi, Seyyed Ali Reza Hashemi, Ayoob Karimi Zade, Ali Mohammad Amani, Amir Savardashtaki, Esmail Mirzaei, Sara Jahandideh, Ahmad Movahedpour

**Affiliations:** a Department of Medical Nanotechnology, School of Advanced Medical Sciences and Technologies, Shiraz University of Medical Sciences, Shiraz, Iran; b Poostchi Ophthalmology Research Center, Department of Ophthalmology, School of Medicine, Shiraz University of Medical Sciences, Shiraz, Iran; c Pharmaceutical Sciences Research Center, Shiraz University of Medical Sciences, Shiraz, Iran; d Department of Medical Biotechnology, School of Advanced Medical Sciences and Technologies, Shiraz University of Medical Sciences, Shiraz, Iran; e Department of Chemical and Polymer Engineering, Faculty of Engineering, Yazd University, Yazd, Iran

**Keywords:** Polyethylenimine, nanocarrier, co-delivery

## Abstract

The meaning of gene therapy is the delivery of DNA or RNA to cells for the treatment or prevention of genetic disorders. The success rate of gene therapy depends on the progression and safe gene delivery system. The vectors available for gene therapy are divided into viral and non-viral systems. Viral vectors cause higher transmission efficiency and long gene expression, but they have major problems, such as immunogenicity, carcinogenicity, the inability to transfer large size genes and high costs. Non-viral gene transfer vectors have attracted more attention because they exhibit less toxicity and the ability to transfer large size genes. However, the clinical application of non-viral methods still faces some limitations, including low transmission efficiency and poor gene expression. In recent years, numerous methods and gene-carriers have been developed to improve gene transfer efficiency. The use of Polyethylenimine (PEI) based transfer of collaboration may create a new way of treating diseases and the combination of chemotherapy and gene therapy. The purpose of this paper is to introduce the PEI as an appropriate vector for the effective gene delivery.

## 1. Introduction

### 1.1. Recent advances in non-viral gene therapy and in vitro/vivo delivery systems

The theory of gene therapy is that treating or preventing genetic disorders by repairing abnormal or replacing the lost genes that have been accelerated over the past few decades as a powerful tool for the treatment of genetic disorders and cancer. A wide field of gene therapy is committed to a number of innovative treatments, which is likely to play an important role in preventing cancer deaths. The success of gene therapy depends on the development of a safe and effective gene delivery system [1,2]. Different methods for gene therapy of cancer using different gene transfer vectors have been studied [3]. In general, the gene delivery systems are classified into viral and non-viral systems. Non-viral systems include (physical: naked DNA, DNA bombardment, electroporation, sonoporation, hydrodynamic, ultrasound, magnetofection, gene gun) and (chemical: cationic lipids, different cationic polymers, cligonucleotides, dendrimers, lipid polymers, inorganic nanoparticles, cell-penetrating peptides) and viral systems include retroviral, adenoviral, adeno association, helper-dependent adenoviral systems, hybrid adenoviral systems, herpes simplex virus, pox virus, lentivirus, epstein-barr virus, cis and trans-acting elements, replication-competent vectors, envelope protein pseudotyping of viral vectors [3,4]. Non-viral vector systems, based on cationic lipids, dendrimers, polymers and peptides have recently been favorable for gene delivery [5], because they are much safer than viral systems that are exposed to immunogenic or inflammatory responses. Viral vectors can achieve higher transduction efficiency and long-term expression of the gene, but they have fundamental problems, such as immunogenicity, insertional mutagenesis, tumorigenicity, capacity and the cost of producing in a large scale [6,7]. Non-viral gene transfer vectors are important compared to viral vectors, for their low toxicity and low preexisting immunogenicity to prevent severe response. In addition, non-viral vectors have a higher loading capacity and quick and easy fabrication [8]. Nano medicine based polymers have attracted a lot of attention due to their high flexibility and various chemical compounds. It is easy to construct a proper structure with chemical and physical properties to provide effective means of drug delivery [9]. The size, shape, surface charge and the presence of different modified functional groups of nanoparticles can affect internal cellular properties (cell-specific internalization), excretion, toxicity and efficacy. The optimal particle size is proposed between 20–200 nm, which is large enough to prevent filtration and small enough to be absorbed through the cell membrane [10]. PEIs with low immune response, degrees of branching and other modifications have been used as reagent transmissions in diverse cell lines and live animals in order to affect small interfering RNA (siRNA) delivery for therapeutic purposes [11]. PEI’s superiority over other polycations such as (L-lysine) is due to high charge density and chain flexibility. PEI electrostatically condenses high molecular weight (MW) DNA to polypeptic nanoparticles (10–100 nm), which are capable of absorbing endocytosis. Both specific target cells and non-specific target cells can endocytose positively charged particles by electrostatic interactions in vitro to achieve high performance in gene delivery. While, in order to function as suitable gene carriers in vivo, the specific absorption efficiency should increase as compared to the nonspecific absorption efficiency. The physical properties of non-viral vector systems, such as the size and potential of zeta play important roles in transmission power.

**Figure 6.**
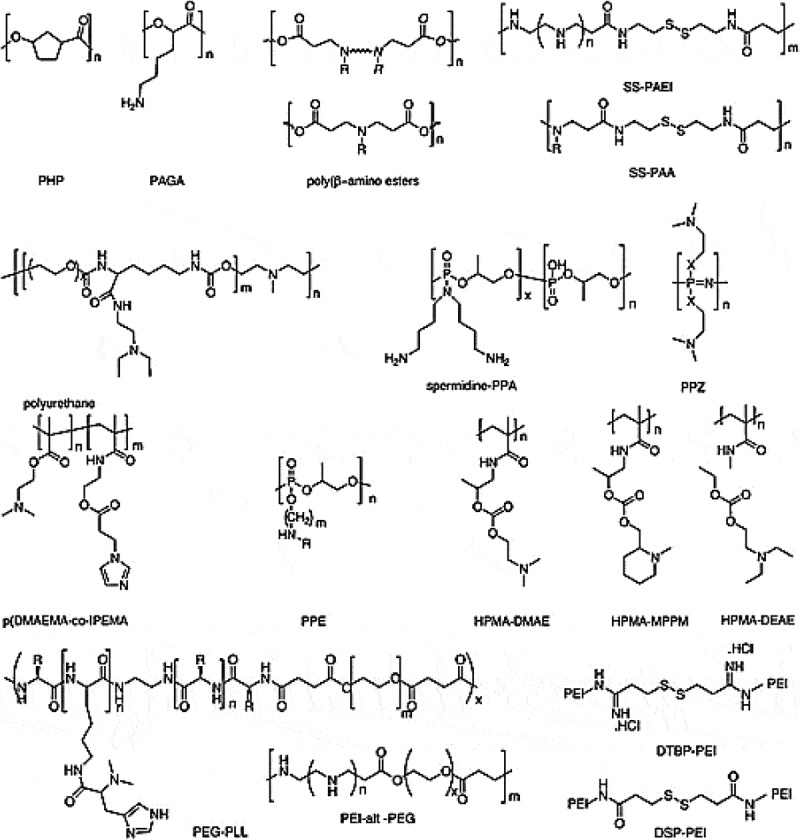
Chemical structures of the degradable cationic polymers [80]. Abbreviations:PHP: Poly(4-hydroxy-L-proline ester); PAGA: Poly(γ-(4-aminobutyl)-L-glycolic acid); SS-PAEI: Reducible poly(amido ethylenimine); SS-PAA: series of linear bioreducible poly(amido amine); PPA: poly(phosphoramidates); PPZ: poly(phosphazenes); p(DMAEMA): poly(dimethylaminoethyl methacrylate); IPEMA: 3-imidazol-1-yl-propionic acid ester of hydroxyethyl methacrylate; PPE: poly(phosphoesters); pHPMA: poly(N-2-hydroxypropyl methacrylamide); DMAE: dimethylaminoethyl; MPPM: 1-methyl-2-piperidine methanol; DEAE: *N,N*-diethylaminoethanol; DTBP: dimethyl 3,3ʹ-dithiobispropionimidate; PEI: poly(ethylene imine); PEG: poly(ethylene glycol); PLL: Poly(L-lysine); DSP: dithiobis(succinimidylpropionate).

## 2. Method

There are currently few attempts to review the current status and the needs of related research. The purpose of this article is to compensate for this shortage by reviewing recent developments and the challenges of PEI in vivo. For this purpose, related articles have been taken on web of science and pubMed sites, and out of 470 applied papers, 35 recent in vivo studies have been selected for further analysis. It is hoped that this article will create new insights and clinical efforts [12]. Over the years, many polymer vectors have been studied. However, the factors that determine their properties (such as DNA compaction capacity, toxicity and transfectability) have not yet been fully elucidated. Among various polycations, PEI has been chosen as an ideal candidate for drug delivery, due to its good efficacy and low cost. However, there is lack of efforts in systematical review of the current status and the unwanted needs of related research. Although the PEI derivatives and their formulations have been investigated. Due to this shortage, this paper evaluated the applications and benefits of the PEI, which were presented separately and remained the only ones written in English. These articles became the main source of information for the overall presentation of PEI applications and further understanding of recent efforts and research progress in this field. The publication of articles between 2005 and 2017, as well as having keywords, were selected for detailed analysis [13]. PEI has been used for many years as a polymer in common processes. Initially, 250 papers were received and 35 articles were selected for more and more accurate examination of PEI results.

## 3. Cationic polymers

### 3.1. Advantages of cationic polymers as nanocarriers

Cationic polymers have attracted great attention in recent years as non-viral vectors in gene delivery due to high cellular uptake efficiency, good water solubility, excellent transferability and easy synthesis. These polymers also have a high potential for drug delivery, which are due to their structure, which is appropriate for the biological process and can easily control the biodistribution and biocompatibility of carrier molecules. Cationic polymers due to their easy synthesis and low cost modification, can be effectively assemble with siRNA through electrostatic interaction and this positively charged condensed complex can improve cellular uptake and siRNA-mediated gene-silencing [14].

### 3.2. Classification of cationic polymers

**Figure 1.**
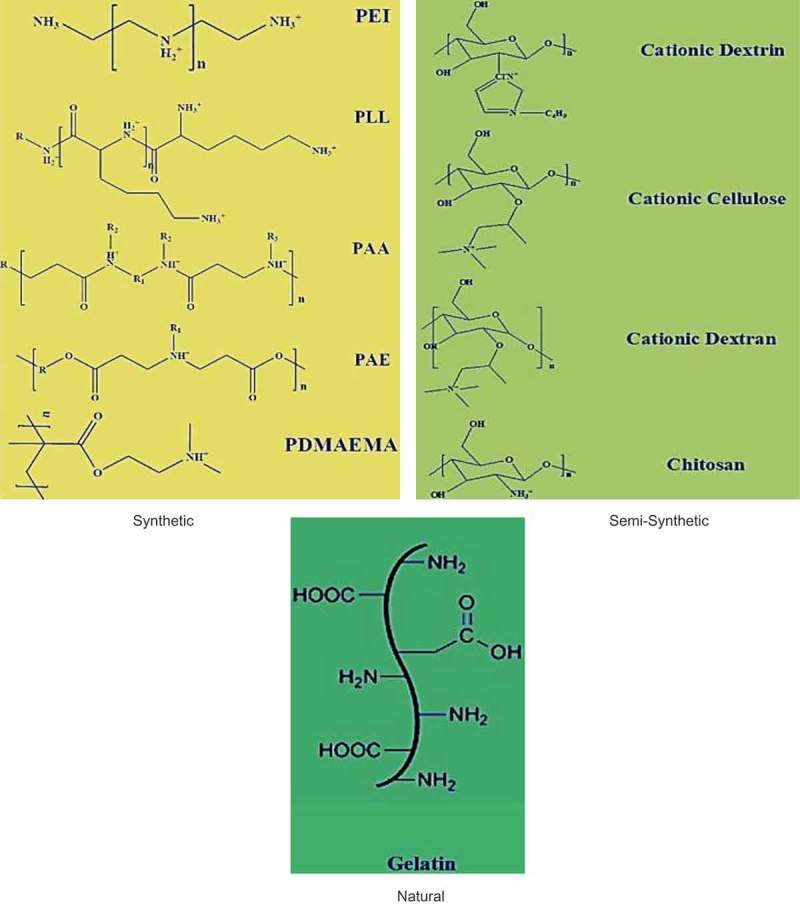
Classification of cationic polymers [14].

Cationic polymers can be referred to positively charged macromolecules that can be inherently present in the polymer backbone or in the side chains. Most cationic polymers have primary, secondary or tertiary amine functional groups that can be protonated. They are also very different in the polymer structure (linear, branched, hyperbranched and dendrimer-like) and can be different by placing positive charges (backbone or side chains) (Figure 1).

## 4. PEI

### 4.1. Structural variation of PEI

**Figure 2.**
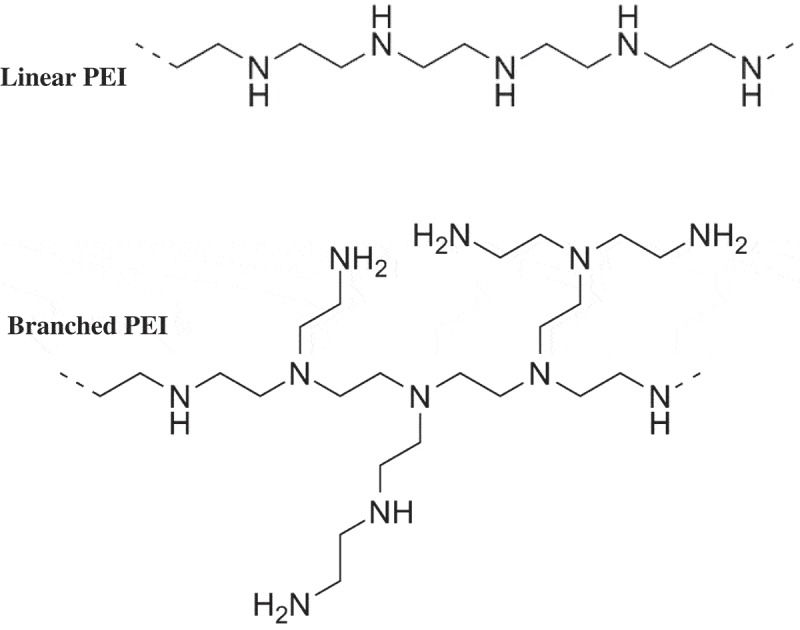
PEI Structure [15].

PEI is a cationic polymer used for many years in typical processes such as paper production, shampoo production, and water purification [15]. However, PEI was not introduced until 1995 as a multipurpose (versatile) vector for gene delivery. Gene therapy is used in different molecular weight, degree of branching, ionic strength of solution, zeta potential and different PEI particle size. PEI is a cationic polymer that is widely used in non-viral gene delivery. However, its widespread use is limited by cytotoxicity. It is assumed that the PEI can have a branched or linear configuration (Figure 2). While the branched PEI (BPEI) has a higher chemical reactivity and can form smaller complexes with DNA in salt-containing conditions, linear PEI (LPEI) is generally less toxic and has the potential for higher transfection efficiency [16]. There are two types of PEI that include linear and branching. In this regard, the branched PEI is formed from ring opening polymerization of aziridine, which contains primary, secondary and tertiary amines. Similarly, the synthesis of linear LPEI is usually carried out via the acid or amine hydrolysis in the backbone of polyoxazoline, which has secondary amines.

Polymer chemistry has new things by business manufacturers, they produce poly(2-ethyl-2-oxazoline) with trade name Aquasolo. In addition, PEI has recently released a high degree of purity for Aquasolo, which is formed from 2-ethyl-2-oxazoline (>99.5% by GC) under compression conditions [17]. On the other hand, PEIs are polymer molecules that consist of repeating amine units and two aliphatic carbon. The branched PEI may have all the primary, secondary and tertiary amino groups, while the linear PEI has only the primary and secondary types. Besides, LPEI is solid at room temperature (near the melting point), while the BPEI is liquid (regardless of molecular weight). In addition, LPEI can be soluble in Boiling taste water with low pH, chloroform, ethanol and methanol [9,18].

**Figure 3.**
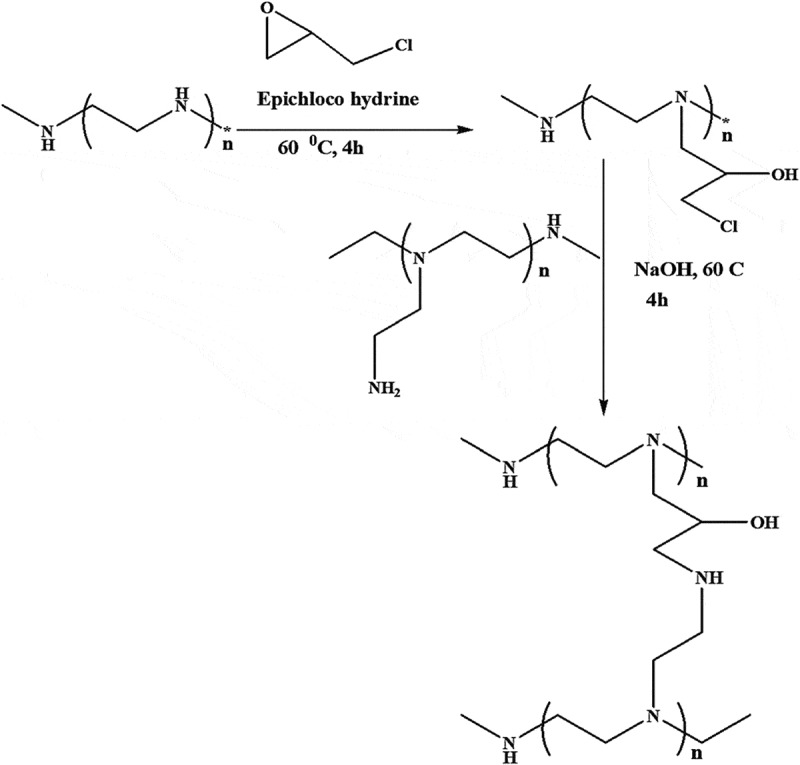
Schematic of BPEI copolymers preparation [20].

In 2016, Itsuno, K. et al., studied low molecular weight branched PEI, linked to linear DNA (600 Da PEI). In this study, it has been shown that separation between heat of binding and heat of condensation is required and the excluded site model should be used for the PEI binding stage in the Isothermal Titration Calorimetry analysis (ITC). They performed the same ITC tests using PEI 25 kDa instead of 600 Da PEI. This means that a large PEI can attach to DNA multivalently, while the small PEI can be linked monovalently [19]. The study of Lungu, C.N., et al. in 2016 on linear and branched PEIs and their spatial characteristics showed that LPEIs modify their geometry easily compared to BPEIs and are more adaptable to a specific binding sites. Overally, PEIs are space-compatible and chemical property space–stable. In general, branched PEIs have a relatively small energetic influence on glucose oxidase enzyme (GOx) in comparison with linear PEIs [18]. In 2016 by Lai, W.F., D.W. Green, and H.S. Jung, a study was conducted on a linear PEI linked with methyl beta-cyclodextrin for the gene delivery. In this study, they linked LPEI with a low molecular weight (which generally showed lower toxicity and higher transfection efficiency) with methyl beta-cyclodextrin (MbetaCD) to form the MbetaCD-LPEI (MLP). MLP shows a slight cytotoxicity in a wide range of concentrations low membrane degradation potential in ex vivo. MLP ensures further developments as a promising gene delivery system for future research [16]. In 2014, Goyal, R., et al., studied copolymers with covalent bonding with linear and branched PEI as efficient nucleic carriers. In this study, linear and branched PEIs with the same molecular weights crosslinked via epoxy sites and their effect on gene delivery ability have been investigated, which seems to result in the production of polymer with lower toxicity and relatively high gene transfection efficiency [20] (Figure3).

In 2013, DeLuca et al. studied on layer composition of ferrocene-modified polyethyleneimine redox polymer films. The significance of these results is due to the first report of the use of the Fc-C6-LPEI redox polymer to improve the success of layer-to-layer composites. The electrochemical reaction obtained with Fc-C6-LPEI/GOX films shows that this redox polymer and layer-to-layer technique can be used for bio-sensor and biofuel applications. Additional applications with enzymatic systems can be the results of the success of multiple anionic polyelectrolytes [21]. In 2010, Koo et al conducted a study on the biodegradation of branched PEI for gene delivery. The degradation of this polymer by multi angle laser light scattering (MALLS) and the electrophoresis gel, and high transfection efficiency and low cytotoxicity were confirmed based on cellular data. Microscopic observations of intracellular trafficking indicate that BPEI does not accumulate in the cell interio [22].

#### 4.1.1. Formulation optimization of PEI-DNA complexes for the efficient gene delivery

Cationic vectors are ideal candidates for gene delivery due to their ability to transfer large genes and scalable production [23]. The synthesis of cationic materials (polymers, lipids) to self-assemble with pDNA based on electrostatic interactions has been significantly improved [24]. PEI cationic polymers have been widely investigated and used as carriers of non-viral gene carriers [25]. Due to its efficacy in facilitating cellular uptake and endosomal escape, which results in increased transfusion efficiency in both in vivo and in vitro, PEI has plenty of superficial positive charges and is able to condense negatively charge DNA into complexes that generate positive charge [26,27]. PEI with a molecular weight of 25 kDa (PEI25k) has been introduced as a ‘gold standard’ for gene carriers [27]. PEI can protect DNA from degradation in the endosomes/lysosomes compartment, due to the ‘proton sponge effect’ [28]. Linear PEI is prepared as an effective carrier for a broad range of gene medicine, including DNA plasmids, small interfering RNAs, mRNAs. In addition, continuous improvement of the physical properties and biological function of polyelectrolyte complex achieved by nanoparticles made of LPEI and nucleic acids [29]. PEI is a polymer that contains amines isolated by ethylene groups and is a hydrophobic because of its rich ethylene-backbone. The PEI chain is charged positively in acidic environments, which is due to secondary amine protonation along the backbone. Because the polymer can absorb H ^+^ ions, it shows weak buffer properties, which are critical to its use as a DNA delivery agent. It protects the DNA from the acidic environment of cell-uptake vesicles and guarantees release of DNA to the cytoplasm [30]. PEI/DNA complexes have shown that they are more stable than their PEI/siRNA counterparts due to the structural differences between DNA and siRNA. The number of interactions in PEI/DNA, which may play an important role in increasing the stability of PEI/DNA complexes [31]. In 2017, Guo, A., et al., worked on the preparation and evaluation of pH-sensitive and rechargeable FA-PEI-CCA/PEI/DNA ternary complex with low toxicity and effective gene delivery (transfer experiments in 293T cells). In this study, modified PEI was obtained with two different levels of NH_2_ replacement, polyethylenimine-1,2-cyclohexanedicarboxylic anhydride (PEI-CCA) and folic-polyethylenimine-1,2-cyclohexanedicarboxylic anhydride (FA-PEI-CCA). PEI-CCA and FA-PEI-CCA have significantly less cytotoxicity and have better blood compatibility than PEI, also have the undifferentiated ability to compress DNA [32]. In 2013, Dempsey et al. discovered that the coating of barium titanate nanoparticles (BT NPs) with PEI improves cellular absorption and is suitable for coupled imaging and gene delivery. BT coating with PEI produced complexes with a zeta-positive potential and increased the BT NPs cellular uptake by 8%. Their major task was to achieve a high level of the gene delivery with the BT-PEI/DNA complexes [33]. Wang et al. proved that bacterial magnetic particles improve the transfer efficiency in mice.

**Figure 4.**
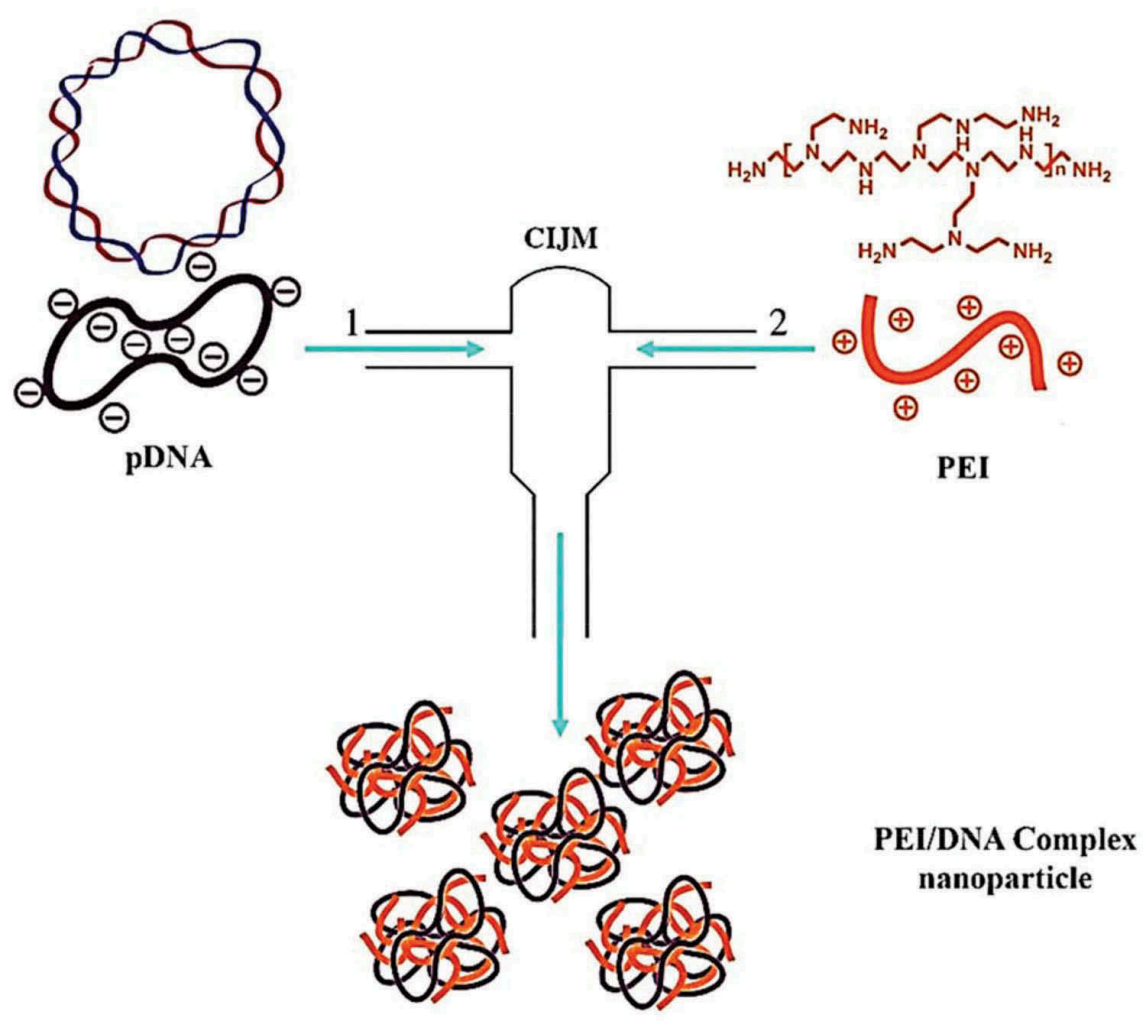
Schematic of the confined impinging jet (CIJ) device used to fabricate polyelectrolyte complex nanoparticles under rapid mixing conditions. (Streams are loaded independently with PEI/DNA plasmid and PEI/DNA nanoparticles, and are placed in a small compartment prior to collection [29].).

In 2017, drug delivery and the effectiveness of BMPPEI complex-conjugated with the foreign DNAs (BPDs) in promoting the testes-mediated gene transfer (TMGT) in mice were studied and the BPDs were compared with the liposome-conjugated foreign DNAs. The results showed that BPD clusters successfully reached cytoplasm and spermatogenesis cell nucleus through the injection test and expressed in the testicle of the transgene founder mice [34]. In 2017, Shih and Venault conducted a study on zwitterionic-shielded carrier with a reversible pH modulation for gene delivery. They exhibited a good design of core-shell polyplexes, which showed PEI as the core and zwitterionic poly(acrylic acid)-block-poly(sulfobetaine methacrylate), ((PAA-b-PSBMA) diblock copolymer) as the shell. Gel retardation tests and displacement of ethidium bromide were used to determine the PEI/DNA or PDMAEMA/DNA complexation. In neutral pH, polymer acts as a protective shell of the complex. Therefore, the results of this study provide a pathway for improving the efficiency and non-toxic properties of pH-sensitive gene carriers [23]. In 2017, Park et al., constructed PLGA nanoparticles coated with Polycistronic SOX5, SOX6 and SOX9 genes for mesenchymal human stem cell chondrogenesis. This multivariate plasmid was complexed with PEI polycationic polymer and poly (lactic-co-glycolic) acid (PLGA) nanoparticles were coated with complex genes (SOX5, SOX6, and SOX9) presented more condrogenic differences than each single gene transfection method [35]. In 2017, Guan et al., study on a pH-responsive detachable polyethyleneglycol (PEG) shielding strategy for gene delivery system in cancer therapy. The primary study endpoints are safety, tolerability and immunogenicity of DNA-PEI vaccination [36]. In 2016, Yang et al., found that the binding of bacterial magnetic particles to PEI increased the gene delivery to breast cells. They reported a method for constructing BMPs-PEI/DNA complexes, which resulted in improved transmission efficiency, reduced cytotoxicity and shorter working time than current methods for formation and transfection of both complexes [37]. In 2016, Santos et al., reported a ﬂash nanocomplexation (FNC) method that continuously produces LPEI DNA/plasmid nanoparticles with a narrow size distribution using a confined impinging jet device. This method involves a combination of negatively charged DNA with positively charged LPEI in fast, high dynamic and homogeneous conditions, which produces complexes of poly-electrolytic nanoparticles with a narrow distribution of particle size and shape [29] (Figure 4).

In 2016, Lazarus et al., reported the synthesis of PEI-coated gold nanoparticles for use as non-viral gene carriers. The results showed that the Au-PEI/pDNA complex increased 12-fold in HEK293 cells and increased 9-fold in HepG_2_ cells compared to PEI/pDNA complex [26].

#### 4.1.2. Parameters affecting PEI-DNA particle size and transfection efficiency

The transfusion efficiency is closely related to the PEI binding with DNA and the ability to overcome a specific barrier [38]. Trafficking and destruction of vectors in lysosomes is one of the main barriers to cellular gene transfer. Akinc et al., have used quantitative methods to study the mechanism of polyethylenimine-mediated DNA transfection. The results are in complete agreement with the proton sponge hypothesis and show that PEI-mediated transfection is effective in avoiding lysosomal trafficking [39]. Polymer/DNA, using a number of possible mechanisms that include DNA protection from enzymatic degradation, faciliting cellular absorption, promoting endolysosomal escape, DNA unpacking in the cytosol, the nucleus and escorting nuclear translocation of DNA or nanoparticles [40]. The excess PEI significantly improves the efficiency of transfection of amounts of polyplex [38]. The transfection process can be affected by many unknown factors. However, several studies have linked the transfection efficiency and cytotoxicity of PEI preparations to the physicochemical properties, the molecular weight and the branching ratio of polymer [41,42], the amount of DNA, the DNA ratio to the PEI, the timing and the solution conditions for complex formation, the transfection medium and cell density at time of transfer [43,44]. In addition, many factors, including temperature, surfactant, complex concentration, ionic strength, viscosity, pH, can significantly affect the aggregation process. Random reduction of particles, increasing electrostatic explosion, preventing particle accumulation and reducing hydrophobicity can make the complex stable [44,45]. Particle size can be mentioned as the important factors affecting the cellular absorption of particles within the cytoplasmic membrane [44,46]. Particle aggregation results from colloidal instability and particle swelling due to charge screening, are two factors contributing to increasing particle size. Salt ions increase the size of PEI-DNA complex and the transfer efficiency, and the longer incubation period has the opposite effect [40,44]. In 2004, Grosse et al., found that cytotoxic effects of PEI could be due to its ability to eliminate endosomes. In fact, this ‘proton sponge effect’ causes high gene transfer capacity. They showed that lactosylated PEI retains the ‘proton sponge effect’ of the polymer unchanged, which is essential for the efficient gene delivery [47].

## 5. Applications of PEI

### 5.1. Medical applications

The delivered gene and the gene carrier determine the non-viral gene delivery strategies [48]. Tissue engineering [48–51], gene therapy [52,53], cancer treatment [54,55], chemotherapy [56,57] and medical applications [58–60] are the applications of PEI.

### 5.2. A developed novel multifunctional delivery system based on PEI for co-delivery

Recently, several delivery systems based on PEI development for cancer treatment have been introduced [61]. Nanocarriers have an important role in delivery of drug-gene combinations to cancer cells [62]. Co-delivery vector based cationic polymer drug has many advantages, which include: 1- stimulate synergistic effect [63], 2- meliorate anti-tumor efficacy and systemic toxicity [64], 3- increase in cytotoxicity and drug absorption in tumor cells [65], 4- improve intracellular bio controllable release [66]. Various types of synthetic particles have been used in drug and gene delivery systems. Among them, PLGA and PEI are the polymers which are being studied more [65]. (Table 1). PEI based co-delivery, may be presented a new method for treating diseases by combination of chemotherapy and gene therapy [61].

**Table 1.** Selected evidence on the therapeutic appeal of poly (ethylenimine) for co-delivery.

Vector	PEI used	Co delivery	Disease model	Properties Therapeutic effects	Ref
hybrid micelles (PCL-PEI) and (PCL-PEG)	PEI2k	Dex or siRNA	Rheumatoid arthritis	Reduce inflammation, without damaging renal or liver function. Thus, blocking NF-kB activation in inflammatory tissue	[61]
cationic pullulan-g-desoxycholic acid-g-PEI	lMbPEI(1kDa)	DOX and P53	MCF-7 cells	Improving antitumor efficacy and systemic toxicity	[64]
poly(EI-co-DCA) copolymers	Linear polyethyleneimine (PEI 423)	Drug demethylcantharate and Akt1 shRNA	(SMMC7721, MCF-7 and A549 cells)	In the present form significantly and synergistically suppress the growth and metastasis of three human cancer cells (SMMC-7721, MCF-7 and A549 cells)	[81]
(FA)-decorated PEG-b-(PCL-g-PEI)-b-PCL triblock copolymers	PEI800	siRNA and doxorubicin (DOX)	Breast cancer cells (MCF7/ADR)	These results proof that PECL3 micelles could co-deliver siRNA and drug to inhibit MDR tumor growth. thus can efficiently reverse MDR cancer and kill the cancer cells	[63]
HA/PEI-PLGA nanoparticles	Linear PEI with the terminal hydroxyl group	Doxorubicin (DOX) and miR-542-3p	TNBCMCF-7 and MDA-MB-231 cells	These nanoparticles have the potential to co-deliver chemotherapeutic agents and tumor suppressive miRNAs in combinatorial TNBC therapy and increased both drug uptake and cytotoxicity in MDA-MB-231 cells	[65]
PEI-hyd-DOX	High molecular weight of 5000 (PEI5k)	pGL-3 plasmid and DOX	HeLa and 293T cells	Proven to be bio-controllably released inside the cells with an enhanced drug efficacy. applicable for the combination of chemotherapy and gene therapy	[66]
PEI and PCL graft copolymer	LMw-PEI	Dox and p53-pDNA	HepG2, Hela and glial tumor	Great potential of the copolymer as an effective platform which provides a stronger cytotoxic effect via higher cellular transfection and simultaneous drug and gene delivery against aggressive cancers	[82]
Two amphiphilic diblock copolymers, (PCL-PEI) and (PCL-PEG)	Branched poly-(ethyleneimine) (MW = 2000, PEI2k)	MicroRNA-34a and Vismodegib	B16F10-CD44+ cells	Candidate delivery vehicle associated with excellent delivery performance and minimal toxicity and great potential in cancer therapy	[83]

Abbreviations: PCL: polycaprolactone, PEI: polyethylenimine; PEG: polyethyleneglycol; Dex: dexamethasone; siRNA: small-interfering RNA; lMbPEI: low-molecular weight branched polyethylenimine; DOX: doxorubicin poly(EI, co-DCA) poly(ethylenimine-co-demethylcantharate); FA: folic acid; TNBC: triple negative breast cancer, HeLa: Human cervix carcinoma cells, 293T: human embryonic kidney transformed 293 cells, -pDNA: plasmid DNA, HepG2: human hepatoma cell line, PLGA: poly (lactic-co-glycolic) acid.

In this study, we present a summary of the design concepts and recent advances for improving drug delivery and gene delivery in vivo and in vitro. These multi-purpose biodegradable nanosystems are ultimately used for cancer chemotherapy and gene therapy [67].

## 6. Cellular toxicity and transfection ability of PEI

**Figure 5.**
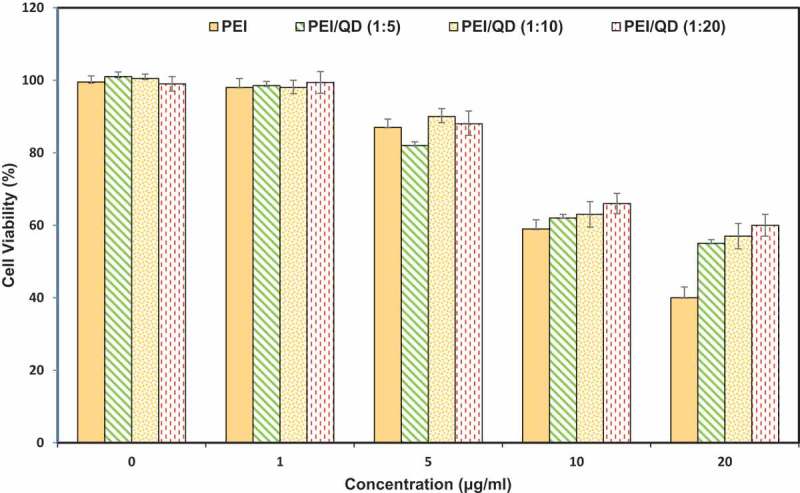
Cell viability and gel electrophoresis assays of different nanocomplexes [77]. Abbreviations: PEI: polyethyleneimine, QD: quantum dot, N/P: nitrogen to phosphate.

Recently, non-viral vectors, which are highly efficient, have been developed by modifying low molecular weight (600 Da) with a variety of functional groups [19]. Because of the high density of the charge and the so-called ‘sponge-proton effect’, PEI has more advantages over other cationic polymers than other non-viral vectors. However, high PEI efficacy is usually associated with high toxicity, which prevents its clinical application, partly due to its nondegradability [68,69]. PEI is a cationic polymer that can be produced in different molecular weights and different configurations such as linear or branching. Linear PEI exhibits less cytotoxicity and higher efficacy than the branched PEI in gene delivery [69]. In general, branched PEI (BPEI) has a greater advantage than linear PEI (LPEI) with a similar molecular weight in binding with nucleic acid and increases molecular absorption while LPEI is superior to BPEI in the releasing/dissecting of nucleic acid [68]. For PEI with different molecular weight, there is a reverse effect (opposite effect) between transfection efficiency and cytotoxicity. Generally, PEI with a higher molecular weight indicates higher transfection efficiency and higher cytotoxicity [69,70]. In addition, damage to the cell membrane reduces the transfection efficiency due to the positive charge of PEI and its non-degradability [71]. PEI is a commercially available gene transfection reagent and is a suitable non-viral vector due to its intrinsic ability to encapsulate genetic material and successfully function in vitro. However, low in vivo transfection efficiency combined with high cytotoxicity limits its further use in gene therapy [72]. Polymer composed of PEI1.8K bonded with acidic bonds can show comparable transfection efficiency compared to high molecular weight PEIs such as PEI25K. Additionally, low endosomal pH (~ 4–5) can break down labile acid PEI to low molecular weight counterparts and reduces the cytotoxicity [73]. Many strategies, such as PEGylation, bonding PEI with functional groups and synthesis of biodegradable PEIs through crosslinking or grafting, have been introduced to reduce cytotoxicity and promote the transfection efficiency of PEI [74]. High positive charge of PEI can result in electrostatic interactions with biological molecules with negative charge (nucleic acids or proteins) and thus facilitate cellular uptake (Figure 5) [75]. However, the non-degradability of PEI has limited its applications in vivo [76]. The non-degradable nature of these polymers has created a serious concern for clinical research, because degradable polymers are more compatible than non-degradable materials. Introducing the reduction-sensitive group to the polymer, which are decomposable by intracellular enzymes, is an interesting approach to the achievement of a degradable, biocompatible non-viral carrier [77].

In 2016, Zhang et al. studied low molecular weight PEI-based vectors with acid-labile ortho ester linkage to enhance the gene delivery. Results showed the improvement of the endosomal process of polyplexes and the reduction of toxicity of the substance after transfusion (post-transfection) [78]. A study was conducted in 2015 by Zhang and coworkers on the preparation of PEI-coated with chitosan and modified with arginine copolymer for DNA delivery. The results show that CS-PEI-Arg derivatives can completely bind pDNA and have complex forms of about 170 nm in size and about 30 mV zeta potential. CS-PEI-Arg has less cellular cytotoxicity than CS-PEI, which is similar to CS and much lower than PEI (Figure 6) [79].

In vitro luciferase analysis shows that CS-PEI-Arg has better transfection efficiency than CS-PEI, which is superior to PEI. The results indicate that CS-PEI-Arg is a prominent candidate for gene delivery and can be used to treat liver or kidney diseases [44].

In 2017, Chen and his coworker developed a biocompatible cationic pullulan-g-desoxycholic acid-g-PEI micelles with average diameter of 160.8 nm and zeta potential of about 28 mV for drug and gene collaboration for cancer therapy (MCF −7). These results showed that the use of a new amphiphilic bifunctional pullulan derivative (named as PDP) micelles is appropriate for effectively co-delivering functional gene and chemotherapeutic agent, which improve the efficacy of antitumor and systemic toxicity and good blood compatibility and low cytotoxicity in the hemolysis [64]. In 2016, Qiu et al. studied the delivery of tanshinone IIA and alpha-mangostin from gold/PEI/cyclodextrin nanoparticle. The size of the nanoparticles was reduced by increasing the molar mass of PEI from 10 to 70 kDa, which was selected to work the designed platform for chemotherapy for prostate cancer. The results of the in vitro preliminary cell viability tests in PC-3 and DU145 prostate cancer cells showed that encapsulation increased cytotoxicity [80].

Very unstable and cerium borohydride reducing reagent which is flammable in the environment may become stable through placing it on a polymeric substrate called polyethylenimine; then, this mild and new reducing reagent which is stable and could be kept for months in laboratory environment may be used in many organic synthetic reactions.Given the large size and the negative charge of nucleic acid, chemical modification of inorganic nanoparticles with functional groups or polymers is generally needed. Positively charged functional groups (usually the amide groups) or polymers, such as amino silane, polyethyleneimine (PEI), polyamidoamine (PAMAM), chitosan, are usually be employed to treat the nanoparticles before binding with DNA. For instance, PEI grafted GO exhibited enhanced chemotherapy efficacy towards HeLa cells by sequential delivery of anticancer drugs and siRNA [81, 82] Aldehydes and ketones are similar in reactivity. The development of methods for the chemo selective reduction of aldehydes in the presence of ketones has therefore received considerable attention or by using additives such as thermoplastic, (PET, ABS, SAN), [83-85] nano tube [86, 87] resins [88, 89], graphen oxide  in Polymer composie  For X-ray radiation shielding [90] and and properties of nanocomposites, linear low density polyethylene, ethylene-co-vinyl acetate and nano clay particles by electron beam [91].

## 7. Conclusion

Gene therapy is capable of treating human diseases caused by defective genes and its purpose is to transfer genetic material to specific cells for the treatment of the disease. Protecting DNA against temperature and pH changes, and the destruction of lysosomes and passing through the membrane to target it are the abilities of the gene as a clinical treatment, which is strongly dependent on the use of appropriate gene directory. Today, all gene delivery carriers have been developed, such as several viral and non-viral vectors, has been improved and used to successfully cure some diseases. In general, both viral and non-viral vectors, despite the advantages and disadvantages, are used in recent advances in gene therapy strategies somewhat bring the expectations of years of gene therapy closer to reality and the hope of achieving more success. It seems that the ultimate gene therapy solution for treating many diseases in this century is human. The result of a great deal of research on this subject in recent years is very promising, and polyethylene imine can be used as an appropriate vector.
